# Minimizing Drug Adverse Events by Informing About the Nocebo Effect—An Experimental Study

**DOI:** 10.3389/fpsyt.2019.00504

**Published:** 2019-07-25

**Authors:** Yiqi Pan, Timm Kinitz, Marin Stapic, Yvonne Nestoriuc

**Affiliations:** ^1^Department of Psychosomatic Medicine and Psychotherapy, University Medical Center Hamburg-Eppendorf, Hamburg, Germany; ^2^Clinical Psychology and Psychotherapy, University of Hamburg, Hamburg, Germany; ^3^Clinical Psychology, Helmut-Schmidt-University/University of the Federal Armed Forces Hamburg, Hamburg, Germany

**Keywords:** nocebo effect, informed consent, patient education, drug safety information, side effects, inert exposure, predictors, risk factors

## Abstract

**Relevance:** Informing patients about potential adverse events as part of the informed consent may facilitate the development of nocebo-driven drug adverse events (nocebo side effects).

**Objective:** To investigate whether informing about the nocebo effect using a short information sheet can reduce nocebo side effects.

**Methods:** A total of *N* = 44 participants with weekly headaches for at least 6 months were recruited using the cover story of a clinical trial for a headache medicine. In reality, all participants took a placebo pill and were randomized to the nocebo information group or the standard leaflet group. Participants were instructed to read the bogus medication leaflet entailing side effects information shortly before pill intake. The nocebo group additionally received an explanation about the nocebo effect as part of the leaflet. Questionnaires were completed at baseline, 2 min, and 4 days after the pill intake. We conducted general linear models with bootstrap sampling. Baseline symptoms were included as a covariate.

**Results:** Most participants (70.5%) reported nocebo side effects at 2 min. Participants who received the nocebo information (*n* = 24) reported less nocebo symptoms than the control group (*n* = 20) (estimated difference: 3.3, BCa 95% CI [1.14; 5.15], *p* = 0.01, Cohen’s *d* = 0.59). Baseline symptoms, perceived sensitivity to medicine, and side effect expectations each moderated the group effect (estimated difference in slope: 0.47, BCa 95% CI [0.19; 0.73], *p* = 0.001, *d* = 0.75; 1.07 [0.27; 1.61], *p* = 0.006, *d* = 0.73; 1.57 [0.38; 2.76], *p* = 0.02, *d* = 0.58). No group differences were found at 4-day follow-up. After revealing the actual aim of the study, 86% of the participants evaluated the nocebo information to be helpful in general.

**Conclusions:** Results provide the first evidence that informing about the nocebo effect can reduce nocebo side effects.

## Introduction

Nocebo effects can cause reduced efficacy of treatments (1, 2) and side effects which are not attributable to the pharmacological or other active ingredients of the treatment (3). Broadly defined, nocebo effects are negative effects caused by psychological and contextual factors of the treatment. As demonstrated in placebo studies (4–6) and in the placebo arms of clinical trials (7–11), side effects are commonly reported after placebo intake. Remarkably, studies which reanalyzed clinical drug trials found considerable overlap in the side effect profiles of drug and placebo arms (7–11). These results indicate that information about potential side effects can influence side effect reporting.

In clinical trials and clinical practice, patients are informed about a treatment’s side effects. However, if information about side effects can increase side effect reporting, does the informed consent potentially undermine the principle of nonmaleficence? Expectations are considered key, given that written and verbal information may lead to increased side effect expectations, which in turn—like a self-fulfilling prophecy—result in more side effects (12–14). Up to now, evidence regarding the effect of side effect disclosure on side effect reporting has been mixed (15). In these studies, patients received the same treatment yet different side effect information. Some studies showed that, the more information patients received, the more side effects they reported (16–19), while others studies found no difference (20–22). Although it cannot be concluded whether informing about side effects is disadvantageous in general, strategies to prevent nocebo side effects may be useful for clinicians, especially when treating patients who are at risk of developing nocebo effects. According to estimates based on adverse events reported in placebo arms of double-blind trials, nocebo side effects account for 40% of drug adverse events across diseases (23). Since adverse events can decrease quality of life, reduce adherence, and, consequently, increase public health costs (24, 25), minimizing nocebo side effects warrants clinical attention.

Researchers have advocated that side effect information should be tailored to the patient to prevent nocebo side effects while maintaining patient autonomy (26). Proposed strategies include permitted noninformation (27), framing (27, 28), and informing about the nocebo effect (3). Permitted noninformation offers patients the possibility of remaining unaware of certain mild side effects. Unlike severe and potentially irreversible side effects, knowledge of less threatening ones is not essential for making an informed choice. The clinician distinguishes between crucial and noncrucial side effect information depending on the treatment indication. Patients then receive a list of side effect categories, and they can decide which category they wish *not* to learn about. Framing, in turn, targets the way in which information is presented. First outlined by Tversky and Kahneman (29), the same probability can be presented either as a gain or a loss, affecting decision making. In clinical practice, the probability of side effect occurrence can either be framed as likely (“40% get a sore arm”) or unlikely (“60% do not get a sore arm”) (30). Some studies have also applied framing in a broader sense; Wilhelm et al. (31) framed dizziness as an onset sensation of the drug, whereas Heisig et al. (32) framed information about potential side effects of breast cancer treatments in the context of expected treatment benefits such as increased survival. The effect of framing on side effects has been investigated in various samples using different experimental methods and has rendered mixed results (30–35).

Barsky and colleagues (3) suggested informing patients about the nocebo effect. When starting a new treatment, most patients have preexisting symptoms due to the natural course of the disease or comorbidities. These baseline symptoms, especially ambiguous ones such as pain, fatigue, and mood swings, can be misattributed to the new treatment. However, if participants are aware that contextual and psychological factors can play a part in the emergence and exacerbation of symptoms, misattribution is less likely to occur (3). Moreover, offering an alternative explanation may result in less attention towards symptoms, thereby reducing its perceived severity (36) and accompanying distress (37). One study examined the efficacy of a nocebo education on symptom reporting. Crichton and Petrie (38) explained symptoms ostensibly caused by infrasound either by a nocebo effect or biological mechanisms and found differences in symptom reporting after an infrasound exposure. Evidence in the clinical context is missing up to now (39).

We aim to investigate the effect of nocebo information on nocebo side effects among persons with weekly headaches. Specifically, we expect participants who receive the nocebo information to report fewer side effects after placebo intake. To understand which participants benefit most from the nocebo information, we will exploratively examine gender (40), perceived sensitivity to medicine (41), anxiety (42), side effect expectations (43), and cognitive coping styles (41) as potential correlates of nocebo side effects and candidate moderators of the hypothesized effect. Except for cognitive coping styles, these factors have been previously linked to nocebo effects (43, 44). As for cognitive coping styles, we presume that a monitoring coping style, i.e., being concerned about potential health threats and being vigilant towards health-related information, is positively associated with nocebo side effects, whereas a blunting coping style, i.e., avoiding confrontation with potentially threatening health-related information, is not. Pronounced monitoring has been associated with increased perception of physical symptoms (45). Given that prior studies found that nocebo effects induced by verbal suggestion can persist for up to 8 days (46, 47), we conducted a 4-day follow-up assessment to examine the time frame of our nocebo induction and of the intervention effect.

## Materials and Methods

### Procedures

In an experimental design, we randomized participants 1:1 to the nocebo information group or the standard leaflet group. We used the cover story of conducting a double-blind phase-IV trial of an already approved headache medication “Relacalmin.” The ostensible aim was to investigate beneficial effects after a one-time intake. Participants were told that they had a 50/50 chance of receiving Relacalmin or a placebo. In fact, all participants received a placebo pill. Except for the 4-day follow-up assessment, which was completed remotely *via* an online link, the study took place at the University Medical Center Hamburg-Eppendorf. Ethical approval was obtained from the ethics committee of the local chamber of psychotherapists (reference number 13/2014-PTK-HH).

Informed consent was signed by all participants before enrolment. Expectations, as well as short- and long-term effects of the medication, were explicitly mentioned in the written informed consent (“A randomization is necessary to underpin whether beneficial effects are caused by an active pharmacological effect or induced by positive expectations;” “It is possible that you will feel better after taking this medicine shortly after intake as well as over the course of four days”).

After signing informed consent, participants completed baseline questionnaires. Then, participants drew from a set of identical looking envelopes. Each envelope contained a medication leaflet and a single blue placebo pill in blister packaging. The nocebo information group and the standard leaflet group received different leaflets. Both leaflets included information about the active substance of the medication, how it works, and its effectiveness (“Studies had shown that head muscle pain is reduced by up to 70%. Participants moreover report an overall feeling of ease and relaxation.”). In line with common medication leaflets, information about contraindications and a list of seven potential adverse events were presented (in the following order): concentration problems, dizziness, vision problems (blurred vision), fatigue, tinnitus, muscle pain, and nosebleed. The adverse events were listed according to their alleged frequency of occurrence from “often,” “sometimes,” to “rarely.” Additional probability information was provided for these frequency specifications, e.g., very often, more than 1 in 10 participants; often, less than 1 in 10 participants, but more than 1 in 100, etc. The nocebo information group received additional information about the nocebo effect as part of the leaflet (**Box 1**). Participants were acquainted with the distinction between specific and nonspecific side effects, and the concepts of misattribution and selective attention. A case example was provided to illustrate the nocebo effect (p. 52f) (48). Written by two investigators (YN and TK), its comprehensibility was evaluated by a self-help cancer patient group and adapted hereafter (39).

Box 1 **Information sheet about nocebo effects.**

**Advance information about side effects**

The occurrence of side effects has two fundamental causes. One cause is the pharmacological (substance dependent) mode of action. Specific pharmacological substances in the drug are metabolized and activate certain biochemical reactions in the body. The second cause is the nonpharmacological (nonsubstance dependent) mode of action. Here, the patient’s expectations and the context of the medication intake activate certain biochemical reactions in the body.

The second cause is labeled the nocebo effect (expectation effect). For example, prior negative experiences or reading about possible side effects in a medication leaflet can increase a patient’s expectations of developing side effects. Consequently, these negative expectations may lead to an actual increase in side effects. The nocebo effect is by no means an illusion; it is a real and measurable response. Clinical studies show that more than half of the experienced side effects can be attributed to expectations.

On the one hand, expectations can lead to actual biochemical changes and, by that, facilitate diseases. On the other hand, expectations can induce heightened awareness of bodily sensations and symptoms. Everyday complaints, which occasionally occur even when no medication is taken, can then be perceived as side effects. Simply expecting illness can lead to actual symptoms. Vice versa, positive expectations can prevent the development of side effects and bring about actual health improvements.

The following example illustrates how expectations emerge and how they affect bodily sensations: “For my next checkup, I was to receive a contrast agent. I was anxious, knowing that my body reacts strongly to that kind of thing. The nurse hooked me up to the IV, through which the contrast agent would enter my body. She told me that the contrast agent would make me feel hot and that there might be a burning sensation. She then left me alone. The minute she left the room, I felt the heat washing over me, it streamed through my body and it burned. I knew this checkup was going to be awful. I felt extremely frightened. After a few minutes, the doctor entered the room and she told me: Ok, let’s inject the contrast agent, shall we?”

Participants were requested to read the leaflet, take the pill, and stay seated for 2 min. Further questionnaires were completed 2 min after pill intake (post). This time frame was chosen to avoid deviations in behavior after intake and to keep nocebo effects, which may be amplified due to symptom monitoring, at a minimum. After completing the questionnaire, participants received an online link for the 4-day follow-up assessment. To match up the questionnaires at post and at 4-day follow-up, participants generated a personal code at enrolment. Interaction between the investigator and the participant was prescripted, neutral, and short (∼5 min in total).

At the 4-day follow-up assessment, participants indicated headache severity, side effects, and what they believed to be the study aim. Afterwards, all participants were debriefed about the actual study aim. Thereby, the nocebo information was presented to all participants. Lastly, the perceived usefulness of the nocebo information was assessed. A reimbursement of 10€ was paid for participation.

### Participants

Eligibility criteria included age ≥18 years and weekly headaches in the past 6 months. To reinforce our cover story, we also added the following exclusion criteria: High sensitivity to pain and fever medication, acute gastrointestinal ulcer, increased risk for bleeding, and severe cardiomyopathy.

### Recruitment

Participants were recruited from the general public in and around Hamburg, Germany, using advertisements in newspapers, online portals, and leaflets distributed in pharmacies and local stores. Screening was conducted *via* phone and, when eligible, an appointment was scheduled.

### Randomization and Blinding

We performed randomization using blocks of eight. After completing the baseline questionnaire, participants were asked to choose one of four opaque, sealed envelopes containing a leaflet (either with or without the nocebo information) and the pill. Depending on the group, the leaflet was labeled either with the letter A or B. The leaflets were otherwise identical (in size and design). Two minutes after taking the pill, participants were asked to state the letter on the leaflet as part of the post assessment. To secure the blinding of the investigator, assessments were conducted using an online form. The investigator sat at a table facing the participant and not the screen. Moreover, the investigator was unaware of the meaning of the letter. All envelopes were prepared before enrolment. The number of prepared envelopes was larger than the required sample size so that every participant was able to choose from a set of envelopes.

### Power Analysis

No previous study has investigated the effect of the nocebo information on side effect reporting. Hence, we have no information on whether the nocebo information is beneficial at all. To keep participants induced with nocebo effects to a minimum, we pragmatically chose the smallest possible sample size. For a one-tailed independent *t*-test, given a large effect size of Cohen’s *d* = 0.8, a power of 0.8, and an alpha error of 5%, we obtained the total sample size of *N* = 42. This sample would allow us to discern whether the nocebo information is useful.

### Measurements

Assessments were conducted at baseline, post, i.e., 2 min after pill intake, and at 4-day follow-up. The questionnaires were identical for both groups. All assessments were conducted using an online form.

#### Cover Story Credibility

The cover story was classified as credible if subjects either reported side effects after 2 min, reported less headache after intake compared to baseline, or expected their symptoms to alleviate after pill intake. At the 4-day follow-up, participants were additionally asked about the goal of the study.

#### Manipulation Check

At post, all participants evaluated the comprehensibility (0 “not comprehensible at all” to 10 “absolutely comprehensible”) of the information in the leaflet. Further questions focusing on the nocebo information were not asked since they might have created suspicion about the cover story.

#### Outcome

Self-reported nocebo side effects were our primary outcome. We use the term nocebo side effects to highlight that, after placebo intake, all reported side effects were nocebo-driven. However, participants—who believed they were taking part in a double-blind trial—were asked about “side effects of the pill.” These were assessed using the validated General Assessment of Side Effects questionnaire (GASE) (49), which we shortened to 20 symptoms, of which 7 were named in the medication leaflet, and 13 were common nonspecific symptoms. Symptoms which were not listed in the leaflet include headache, hair loss, dry mouth, circulation problems, abdominal pain, nausea, diarrhea, skin rash or itching, fever/increased temperature, tendency to develop bruises, insomnia/sleeping problems, back pain, and irritability/nervousness. We did not exclude headache from the symptom list since it has been previously reported as an adverse event in headache trials (50). Participants were instructed to indicate only the symptoms they attributed to the pill. Each symptom was rated on a scale from 0 “not present,” 1 “ mild,” 2 “moderate,” to 3 “severe.” Sum scores were composed for total nocebo side effects, nocebo side effects which were listed in the leaflet (leaflet nocebo side effects), and nocebo side effects which were not listed in the leaflet (nonlisted nocebo side effects). Additionally, we also calculated the total number of nocebo side effects. This questionnaire was administered at 2 min after intake (post) and at 4-day follow-up.

#### Potential Predictors of Nocebo Side Effects, Expectation Change

All potential predictors were assessed at baseline.

**Baseline symptoms.** We used the same shortened GASE questionnaire to assess the number and severity of symptoms in the past 4 days. A sum score with a range of 0–60 was calculated.

**Perceived sensitivity to medicine.** Five items assessed the “belief that one is especially sensitive to the actions and side effects of medicine” (p. 1) (41) on a scale from 1 “strongly agree” to 5 “strongly disagree.” The items were reversed and a sum score was computed, ranging from 5 to 25. The validity and reliability have been shown among different patient groups as well as among healthy participants (51).

**Trait Anxiety.** The State-Trait Anxiety Inventory is a commonly used instrument with good psychometric properties (52). We used the trait scale only. Twenty items are rated on a scale from 1 “almost never” to 4 “almost always.” A sum score is obtained and ranges from 20 to 80.

**Cognitive coping mechanisms.** The Threatening Medical Situation Inventory assesses the degree to which individuals cope with threatening information by confronting and seeking out further information (monitoring, e.g., “I plan to ask the specialist as many questions as possible”) or by avoiding information (blunting, e.g., “I think things will turn out to be alright”) (53). We presented participants with two of the four possible medical scenarios (headaches and appendicitis) which included six items, respectively. Mean scores range from 1 to 5. The validity and reliability have been established previously (53).

**Sociodemographics.** Age, years of education, and gender were assessed with the latter investigated as a potential predictor of nocebo side effects.

**Expectations.** Participants indicated to which extent they expected the occurrence of side effects on a scale from 0 (absolutely disagree) to 10 (absolutely agree). Two filler items for the cover story inquired about subjects’ expectations of headache reduction and their overall treatment expectations. Expectations were assessed at baseline and post. This would allow us to explore whether expectations changed overall and whether the change varied by group.

#### Placebo Effect, Evaluation of the Nocebo Information

**Headache.** At baseline, post, and 4-day follow-up, participants specified their current intensity of headache, state of relaxation, and overall well-being on a scale from 0 (none) to 10 (highest imaginable), with the latter two items being filler items. Placebo effects were operationalized as the difference in headache between baseline and post. Inquiries about symptom amelioration of symptoms at 4-day follow-up were filler items to balance out inquiries about side effects; no computation of 4-day placebo effects was performed since disentanglement from the natural course of the disease was not possible.

**Evaluation of the nocebo information.** After debriefing about the true study aim and presenting the nocebo information to all participants at 4-day follow-up, participants were asked whether they consider informing about the nocebo effect to be useful in general (yes/no).

### Statistical Analyses

To assess whether nocebo side effects at post differed between the groups, we conducted general linear models (GLM) using the maximum likelihood estimation method. We adjusted for baseline symptoms since they are a confounder of our outcome (54). Except for the estimation method of parameters, GLM aligns with multiple linear regression models. To account for violations of heteroscedasticity, standard errors and 95% confidence intervals (CI) were obtained through nonparametric bootstrap resampling (55) with 2,000 replications and bias-corrected and accelerated (BCa) intervals. Further assumptions including the normal distribution of residuals and no multicollinearity of predictors were checked and met. If univariate associations were given between nocebo side effects and personality characteristics, baseline symptoms, expectations, or gender, moderation analyses were computed (56, 57). To obtain effect sizes, we divided the mean group difference by the standard error of the group difference multiplied by the square rooted number of participants in the standard leaflet group (58). Baseline symptoms were centered and included as a covariate in all models. For moderation analyses, the centered moderator variable and the product of moderator by group were included additionally. To determine the predictive value of the moderation effect, likelihood ratio tests in comparison with the intercept-only model were conducted.

Further analyses were performed to outline the placebo effect, the change in side effect expectations from baseline to post, and whether nocebo side effects sustained up to 4 days. Group differences in nocebo side effects at 4-day follow-up were examined using GLM after adjusting for baseline symptoms. Since associations between nocebo responders and placebo responders have been found previously (59), and since participants may view side effects as onset symptoms of the drug (60), which again, may facilitate placebo effects, correlations between headache change from baseline to post and nocebo side effects at post were investigated. Analyses were performed using IBM SPSS Version 25; GLMs were computed using the GENLIN command. All tests were conducted two-sided with an alpha error of 0.05.

## Results

Baseline characteristics of the sample are portrayed in **Table 1**. The sample consisted mainly of women (70.5%), and most participants had at least a high school degree (88.6%). Participants reported an average of 9 (SD = 4.2) baseline symptoms. Most participants (*n* = 38; 86.4%) had a headache at baseline of an averaged mild to moderate severity (*M* = 3.3, SD = 2.5). The groups did not differ considering baseline characteristics. The cover story was credible, since all participants either expected headache reduction, experienced a headache reduction at 2 min, or reported nocebo side effects after 2 min. Both groups evaluated the leaflet information to be very comprehensible (nocebo information group: *M* = 9.1, SD = 1.6; control group: *M* = 9.4, SD = 1.5). When inquired about the study goal, almost all participants (95.5%) specified an answer in alignment with the cover story (e.g., “whether the medication works,” “side effects of the drug,” or “time course of drug efficacy” etc). Only two individuals indicated “placebo effect.” Although it is not evident what they meant, it is possible that they questioned the cover story. Sensitivity analyses were conducted after exclusion of these two participants.

**Table 1:** Sample characteristics.

	Total sample (*N* = 44)	Nocebo information (*n* = 24)	Standard leaflet (*n* = 20)	Group comparison
	Mean (SD)	Mean (SD), Range	Mean (SD), Range	
**Demographic and clinical information**				
Age, years	30.7(11.2)	31.4(9.1) 18–52	30.0(13.2) 18–65	*t*(42) = 0.4; *p* = 0.68
Female; *n* (%)	31(70.5)	17(70.8)	14(70.0)	*p* = 1.00, FET
≥ 13 years of education; *n* (%)	39(88.6)	22(91.7)	17(85.0)	*p* = 0.65, FET
Baseline symptoms sum score (intensity × numbers)	13.55(7.8)	14.00(6.7) 3–30	13.00(9.0) 3–33	t(42) = 0.4; p = 0.68
Number of baseline symptoms	9.1(4.2)	9.4(3.8) 2–17	8.65(4.8) 2–17	*t*(42) = 0.6; *p* = 0.55
Current headache severity^a^	3.3(2.5)	3.4(2.1) 1–8	3.2(2.3) 1–8	*t*(36) = −0.2; *p* = 0.83
**Personality characteristics**				
Perceived sensitivity to medicine	8.9(3.7)	8.8(4.2) 5–18	9.1(2.9) 5–16	*t*(42) = 0.2; *p* = 0.82
Trait anxiety	45.2(12.3)	44.0(11.8) 24–76	46.7(13.0) 29–65	*t*(42) = 0.7; *p* = 0.47
Monitoring cognitive coping style	3.3(0.6)	3.3(0.6) 1.7–4.2	3.4(0.6) 2.2–4.5	*t*(42) = 0.4; *p* = 0.72
Blunting cognitive coping style	3.2(0.7)	3.3(0.7) 2.0–5.0	3.0(0.7) 1.5–4.2	*t*(42) = −1.2; *p* = 0.23
**Expectations**				
Expectations about side effect occurrence^b^	2.2(1.9)	2.4(2.0) 0–6	2.1(1.8) 0–6	*t*(42) = −0.6; *p* = 0.58

SD, standard deviation; T, Student’s t-test for independent samples; FET, Fisher’s exact test.

a Indicated for n = 38 persons (nocebo information: n = 20; standard leaflet: n = 18) who suffered from headache at the time of baseline assessment, i.e., reported a score of 1 or higher. Headache severity was rated from 0 (no pain) to 10 (worst imaginable pain).

b Expectation about side effect occurrence was rated on a scale from 0 to 10.

### Nocebo Side Effects

At 2 min after intake, 31 (70.5%) participants reported at least one symptom. The most reported symptoms were headache (56.8%), dry mouth (29.5%), exhaustion (29.5%), vision problems (22.7%), back pain (22.7%), and irritability (22.7%). Out of 20 possible side effects, 41.7 and 15% of participants in the nocebo information and standard leaflet group, respectively, reported no symptoms.

According to generalized linear models with bootstrap sampling, participants in the nocebo information group reported less nocebo side effects (sum score) after 2 min compared to participants in the standard leaflet group (**Table 2**). Baseline symptoms predicted nocebo side effects (*B* = 0.47, BCa 95% CI [0.27; 0.63], *p* < 0.001). The group difference remained when headache was excluded from the list of nocebo side effects (estimated difference: 3.2, BCa 95% CI [0.98; 5.07], *p* = 0.02, Cohen’s *d* = 0.56) and after exclusion of two participants who may have questioned the cover story (3.4, BCa 95% CI [0.81; 5.67], *p* = 0.01, Cohen’s *d* = 0.60). When nocebo side effects presented (7 symptoms) and not presented in the leaflet (13 symptoms) were analyzed separately, group differences were found only for nonlisted nocebo side effects, yet not for leaflet nocebo side effects. Individuals in the nocebo information group reported an estimated 2.8 (BCa 95% CI [1.0; 4.4], *p* = 0.009, Cohen’s *d* = 0.66) fewer nocebo symptoms.

**Table 2:** Nocebo side effects by intervention group.

	Group	Unadjusted Mean (SE)	Estimate^a^ (SE)	Estimated difference (SE)	BCa 95% CI		*p*	Cohen’s d^b^
					Lower	Upper		
Sum^c^	Nocebo	3.00 (0.84)	2.79 (0.79)	3.28 (1.24)	1.14	5.15	0.01*	0.59
	Standard	5.80 (1.47)	6.05 (0.86)					
Leaflet	Nocebo	1.25 (0.41)	1.09 (0.34)	0.85 (0.51)	−0.11	1.91	0.10	0.37
	Standard	1.75 (0.49)	1.94 (0.37)					
Nonlisted	Nocebo	1.75 (0.52)	1.73 (0.51)	2.34 (0.85)	1.04	3.43	0.004**	0.62
	Standard	4.05 (1.06)	4.07 (0.56)					
Number	Nocebo	2.38 (0.61)	2.15 (0.64)	2.77 (0.94)	1.03	4.39	0.009**	0.66
	Standard	4.65 (1.11)	4.92 (0.58)					

N, 44; SE, standard error; BCa, bias-corrected and accelerated; CI, confidence interval; Nocebo, nocebo information group; Standard, standard leaflet group.

a Estimates of general linear models with bootstrap sampling (2,000 samples), adjusted for baseline symptoms held constant at its mean.

b Mean estimated group difference/(standard error of the estimated group difference * √ sample size of the standard leaflet group).

c A list of 20 symptoms were presented, of which 7 were portrayed as bogus side effects in the leaflet, and 13 were common side effects of medications (nonlisted). The severity of each symptom was rated as 1 “mild,” 2 “moderate,” or 3 “severe.”

*p < 0.05, **p < 0.01

### Predictors of Nocebo Side Effects and Moderators of the Intervention

Nocebo side effects correlated significantly with baseline symptoms (*r* = 0.64, *p* < 0.001), a monitoring cognitive coping style (*r* = 0.32, *p* = 0.04), and trait anxiety (*r* = 0.47, *p* = 0.001), and in trend with perceived sensitivity to medicine (*r* = 0.29, *p* = 0.06), and side effect expectations (*r* = 0.28, *p* = 0.07). No associations were found with a blunting cognitive coping style (*r* = −0.15, *p* = 0.33) or gender (*r* = 0.18, *p* = 0.24). Among the predictors, we found that baseline symptoms correlated with perceived sensitivity of medicine (*r* = 0.30, *p* = 0.049), trait anxiety (*r* = 0.55, *p* < 0.001), and side effect expectations (*r* = 0.34, *p* = 0.02). All the other variables were not associated.

**Figure 1:**
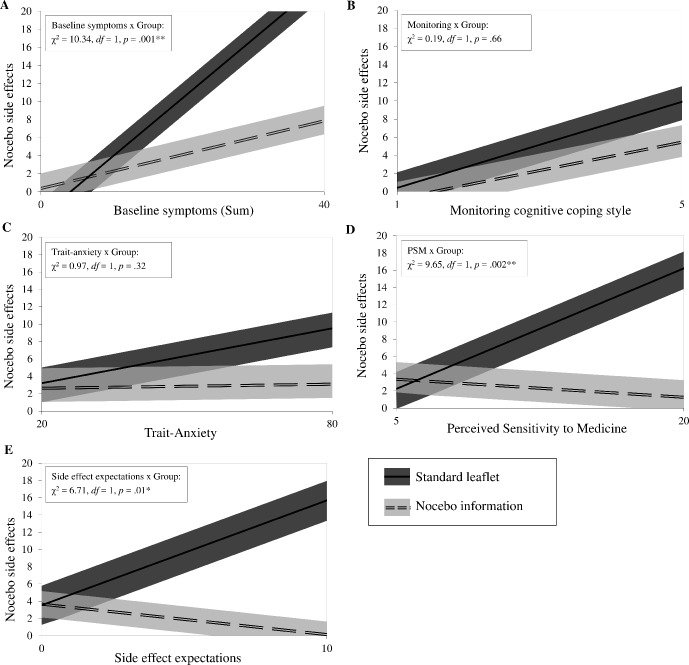
Moderators of the intervention. The panels **(A** to **E)** show the candidate moderators on the x-axis: baseline symptoms, monitoring cognitive coping style, trait-anxiety, perceived sensitivity to medicine, and side effect expectations. For each panel, the primary outcome nocebo side effects is shown on the y-axis.The relationship between each moderator and nocebo side effects by intervention group (nocebo information: *n* = 24; standard leaflet: *n* = 20) are presented using estimates of general linear models with bootstrap sampling, adjusted for baseline symptoms. Bias-corrected and accelerated 95% confidence intervals are portrayed as upper and lower boundaries. For interaction effects, log-likelihood tests comparing each model with the intercept-only model are shown in the upper left area. *p < 0.05, **p < 0.01.

Baseline symptoms, a monitoring cognitive coping style, trait anxiety, perceived sensitivity to medicine, and side effect expectations were further examined as moderators of the group effect (**Figure 1**). Baseline symptoms x group added predictive value over and above the intercept-only model (*χ* ^2^ = 10.34, *df =* 1, *p* = 0.001). The slopes between the groups differed significantly (estimated mean difference = 0.47, BCa 95% CI [0.19; 0.73], *p* = 0.001, Cohen’s *d* = 0.75), indicating that, with increased baseline symptoms, nocebo side effects also increased. This effect, however, was buffered by the nocebo information. The same pattern was found for perceived sensitivity to medicine (1.07, BCa 95% CI [0.27; 1.61], *p* = 0.006, Cohen’s *d* = 0.73) and side effect expectations (1.57, BCa 95% CI [0.38; 2.76], *p* = 0.02, Cohen’s *d* = 0.58). Trait anxiety and a monitoring cognitive coping style did not moderate the effect of the intervention.

### Placebo Effects, Expectation Change, Sustained Nocebo Side Effects

Six (13.7%) participants reported reduced headache compared to baseline, indicating that the placebo effect after 2 min, if at all existent, was marginal. Hence, we did not examine the link between headache change and nocebo side effects.

Overall, side effect expectation change from baseline to post was marginal (*M* = 0.23, SD = 1.05). Expectation change did not differ by group [*M*_Nocebo information_ = 0.33; SD = 1.12; *M*_Standard leaflet_ = 0.10; SD = 0.97; *t*(42) = 7.3, *p* = 0.47].

*N* = 42 participants completed the 4-day follow-up assessment. A total of *n* = 41 (97.6%) participants reported at least one nocebo side effect. Participants in the nocebo information group (*n* = 22) and the standard leaflet group (*n* = 20) reported nocebo side effect sum scores (intensity × numbers) of *M* = 8.2 (SD = 8.8) and *M* = 9.0 (SD* =* 7.2). An averaged number of *M* = 5.7 (SD = 5.1) and *M* = 6.4 (SD = 4.6) nocebo side effects were indicated, respectively. No group differences were found for the side effect sum score at 4-day follow up (estimated difference: −0.42, BCa 95% CI [−3.22; 2.11], *p* = 0.78).

### Evaluation of the Nocebo Information

After participants were debriefed about the true study goal, most of them (*n* = 36, 85.7%) considered the nocebo information to be useful in general. Five participants wrote additional comments with regard to its usefulness. One person wrote: “For me, it [the nocebo information] had no effect because I read the potential side effects only briefly. But now I remember that I had an earache which made me remember the side effect tinnitus. I had a pretty strong headache and thought, if I really had taken medication, this one did not work at all, yet the side effects did affect me.” Another person wrote, “I would have believed the same thing [referring to the case example in the nocebo information], because I am a little anxious.” Three individuals referred to the nocebo information as “interesting.”

## Discussion

The present findings suggest that participants with weekly headaches report less nocebo side effects when they were previously informed about the nocebo effect. In this experimental, ostensibly double-blind medication study, we have found that after placebo intake, individuals who received a one-page nocebo information sheet embedded in the medication leaflet reported an averaged 2.8 (95% CI [1.0; 4.4]) fewer symptoms compared to patients who solely received the medication leaflet. Nocebo side effects were significantly associated with heightened baseline symptoms, trait anxiety, and a monitoring cognitive coping style, and in trend with perceived sensitivity to medicine, and side effect expectations. No associations were found with a blunting cognitive coping style or gender. Explorative moderation analyses indicate that the beneficial effects of the nocebo information are more pronounced among participants with high rates of baseline symptoms, participants who perceived themselves to be highly sensitive to medication, and participants who were more confident that they would develop side effects.

Novel treatments may trigger an individual’s attention towards potential meaningful symptoms—an essential procedure in order to initiate corresponding health behavior, e.g., side effect treatment and coping, or as in double-blind trials, for detailed recording of adverse events to evaluate treatment safety. Barsky (3, 61) proposed that nocebo side effects emerge when everyday complaints are misattributed as side effects. These symptoms, again, can be amplified through the individual’s selective attention towards bodily signals. The nocebo information provides a framework which allows for a more benign interpretation of symptoms and, by that, breaks the vicious circle of amplification. Although due to the inert treatment in our study, we cannot evaluate whether symptom amplification can be prevented, yet we have shown that the additional information may help reduce symptom misattribution.

As implied in Barsky’s theory, and in alignment with a number of empirical studies (43, 62), some patients appear to be more prone to developing nocebo side effects than others. Etiological models on symptom exacerbation through psychological factors postulate that patients with health worries and generally higher anxiety tend to engage in selective interoceptive awareness (37). This is reflected in our findings; participants with increased trait anxiety developed more nocebo side effects. This link has also been found in other studies (33, 59, 63). A monitoring cognitive coping style, which on the other hand has never been investigated in the context of nocebo effects, predicted nocebo side effects as well. “Monitorers” seek to gather as much information as possible about health risks. We propose that both procedures—monitoring health information and monitoring bodily signals—originate from the same motivational goal of gaining reassurance. It is therefore likely that certain patients score high on both characteristics. In accordance with this reasoning, we found that a blunting cognitive coping style, i.e., avoiding information in face of medical threats, was not associated with nocebo side effects. Lastly, we found a high correlation between nocebo side effects and baseline symptoms. Patients with more baseline symptoms have a larger “pool” of symptoms of which they might identify as a side effect. In summary, patients who have many baseline symptoms, are more anxious, or tend to seek out information when facing potential health threats are more vulnerable to developing nocebo side effects.

In contrast to previous studies (33, 40, 64), we did not find an association between female gender and nocebo side effects. However, our sample size was small, and the proportion of female participants was high (70.5%), which does not allow for conclusions in this regard.

Notably, the nocebo information did not buffer the effect of trait anxiety and monitoring on nocebo side effects. It did, however, buffer the effects of baseline symptoms, perceived sensitivity to medicine, and side effect expectations on nocebo side effects. A link between perceived sensitivity to medicine and side effects, and a link between side effect expectations and side effects have been found in previous research (12, 13, 41, 65). In this study, these associations constitute only a trend. The predictive coding paradigm suggests that prior information generate predictions which, in turn, cocreate perception (66, 67). Thereby, sensory input is more likely to be perceived in line with predictions. Henningsen and colleagues suggested that enabling more precise predictions would facilitate a more differentiated perception of bodily sensations (66). Both side effect expectations and perceived sensitivity to medicine, which is characterized by agreeing to statements like “My body overreacts to medicines” or “Even small amounts of medicine can upset my body,” are predictive of side effect development. We believe that, by distinguishing between specific and nonspecific side effects in the nocebo information, participants limited their predictions about side effects to the symptoms mentioned in the leaflet. This suggestion is corroborated by the finding that the groups differed only with regard to the side effects which were not listed in the leaflet, but not those which *were* listed. Interestingly, the specification of prediction was not reflected in a change of side effect expectations. Since the term side effects usually refers to pharmacological side effects, we presume that patients recognize nocebo effects to be, by definition, no side effects. In other words, knowing that symptoms can be misperceived as side effects and therefore intensify is, from the patient’s perceptive, unrelated to pharmacological side effects and corresponding expectations.

The overall rate of nocebo response (70.5%) was higher compared to previous clinical trials. Adverse event rates following placebo intake amount to 18.4–18.7% for the acute treatment of migraine and cluster headaches and 24.0–42.8% for the preventive treatment of migraine and tension-type headaches (8). Mitsikostas et al. (9) have argued that high nocebo response rates reflect a more burdened patient population since comorbidities such as somatization and anxiety are more common among chronic headache patients. Indeed, a US survey with migraine patients found depression (63.8%), anxiety (60.4%), chronic pain (39.5%), and irritable bowel syndrome (29.3%) to be the most common comorbid conditions (68). However, whether or not this rationale is applicable to our patient sample cannot be confirmed due to the lack of diagnostic information. The discrepancies to other studies may also arise from different methods of adverse event assessment. Several reviews have pointed out inadequate reporting of adverse events in clinical trials (69, 70). It is common that assessments consist of open-ended questions from the investigators and spontaneous reports of participants, which leads to lower side effect reports compared to a systematic assessment of side effects as used in this trial.

At the 4-day follow-up, 97.6% of participants reported nocebo side effects. These reports did not differ by group. In line with these findings, a recent study showed that framing of side effect information reduced nocebo side effects short term but not after 24 h (33). However, we did not induce nocebo effects after 4 days due to ethical reasons but suggested a potential positive effect of the medication for 4 days. Consequently, some participants might have perceived side effects after 4 days to be unlikely. Given that the nocebo side effect sum scores at the 4-day follow-up were strikingly high compared to post-intake (difference by 4.1 points), it is uncertain whether some participants might have simply specified all of their symptoms, irrespective of whether they were attributed to the pill. Conclusions about the persistence of an indirect nocebo induction, i.e., through a leaflet and without verbal suggestions of symptom worsening, and the mid- or long-term beneficial effects of the nocebo information cannot decisively be drawn from our data. Further studies are warranted to this end.

### Limitations

This study has a number of limitations due to its pilot character. The sample size is small; although we conducted interaction tests which are recommended to assess differential subgroup effects (56), the moderation analyses, in particular, are based on a modest number of participants. These results should be viewed as hypothesis-generating and necessitate further evaluation in future studies. In addition, the sample size calculation was based on a Student’s *t*-test for independent samples, yet main analyses were conducted after adjustment for baseline symptoms. Given that after inclusion of a covariate, a bigger sample size might have been necessary, our sample size estimation was liberal. The time points of 2 min and 4 days were chosen based on ethics and prior research on nocebo effects and do not align with the onset and duration of actual headache medications. In other words, studies which ostensibly administer medications do not give suggestions into a “vacuum” but rather trigger expectations related to the patients’ prior experiences. Common headache drugs reach maximum plasma concentration 30–120 min after intake (71), whereas assessment after 2 h is a gold standard in headache trials (72, 73). Therefore, the direction of bias is unknown. On the one hand, nocebo side effects may be underestimated due to the short time period of 2 min. On the other hand, the short time frame may have promoted cognitive availability of the nocebo information and resulted in an overestimated influence of the intervention. In addition, patients in headache trials are instructed to take the medication when experiencing acute symptoms. In our study, six participants did not have a headache at the time of pill intake. In light of this, placebo effects at post were marginal. However, this does not necessarily signify unreliable reports of nocebo side effects. Prior evidence has shown that nocebo effects are elicited more easily than placebo effects (59, 74). Nonetheless, matching assessment points to the duration of effect of available medication and facilitating placebo effects could render more precise estimates of nocebo side effects and of the intervention effect, also with regard to its sustained effects.

It should be noted that our findings—although potentially highly relevant—cannot be transferred into clinical practice. In contrast to clinical practice, all participants took a placebo instead of an active medication. Moreover, they believed that they were taking part in a drug study, i.e., had a 50/50 chance of receiving either the medication or the placebo. This context differs from clinical practice, in which patients have 100% certainty of receiving treatment. Again, the direction of bias is unknown. Nocebo side effects could have been underestimated if participants believed to be in the placebo arm. They could also have been overestimated since uncertainty about safety and group affiliation can result in increased monitoring of symptoms. Lastly, given our liberal inclusion criteria (weekly headaches for at least 6 weeks), we cannot determine our sample considering headache diagnoses and comorbidities. It is probable that our study included both individuals with episodic and chronic headache types. Differential subgroup effects by diagnoses cannot be investigated.

### Implications

This study provides the first evidence that informing about the nocebo effect may be a viable strategy for reducing nocebo side effects. The strengths of the nocebo information consist of its convenience and feasibility; a standardized, short information sheet can be handed out by practitioners or pharmacists as an add-on to a new medication. However, due to its limitations, this trial should be perceived as a proof-of-concept. To determine the value of the nocebo information, further trials in clinical practice, i.e., with clearly specified patient groups undergoing active treatments, are needed.

## Ethics Statement

This study was carried out in accordance with the recommendations of the Ethics Commission of the Chamber of Psychotherapists in Hamburg with written informed consent from all subjects. All subjects gave written informed consent in accordance with the Declaration of Helsinki. The protocol was approved by the the Ethics Commission of the Chamber of Psychotherapists in Hamburg. English translation of the ethics statement: Application number: 13/2014-PTK-HH Research project: “Can a patient education reduce side effects? An Experimental Study on the Nocebo Effect” Dear Prof. Dr. Nestoriuc, The Ethics Commission of the Chamber of Psychotherapists in Hamburg has issued the following statement after examining the documents submitted by you in order to examine the compatibility of the given study with ethical principles: After reviewing the documents submitted by you as the responsible head of the study on the aforementioned research project dated 10 September 2014, the Ethics Commission of the Chamber of Psychotherapists in Hamburg came to the conclusion that there were no ethical objections to study conduction. Based on this statement, we can inform you that there are no objections to the conduct of the study. Yours sincerely, Prof. Dr. Hertha Richter-Appelt Chairwoman of the Ethics Committee.

Original in German: Antragsnummer: 13/2014-PTK-Hamburg Forschungsvorhaben: Kann eine gute Aufklärung Nebenwirkungen reduzieren? Eine experimentelle Studie zum Nocebo-Effekt". Sehr geehrte Frau Prof. Dr. Nestoriuc, die Ethikkommission der Psychotherapeutenkammer Hamburg hat nach Prüfung der von Ihnen vorgelegten Unterlagen auf Prüfung der Vereinbarkeit der im Rubrum genannten Studie mit ethischen Grundsätzen die folgende Stellungnahme abgegeben: Nach Sichtung der von Ihnen als verantwortlicher Studienleiterin eingereichten Unterlagen zu dem vorgenannten Forschungsvorhaben vom 10.September 2014 ist die Ethikkommission der Psychotherapeutenkammer Hamburg zu dem Ergebnis gekommen, dass der Durchführung der Studie keine ethischen Einwände entgegenstehen. Aufgrund dieser Stellungnahme können wir Ihnen mitteilen, dass der Durchführung der Studie keine Einwände entgegenstehen. Mit freundlichen Grüßen, Prof. Dr. Hertha Richter-Appelt, Vorsitzende der Ethikkommission.

## Author Contributions

YN and TK initiated the study design. TK and YP conducted the study. YP and MS analyzed and interpreted the data. YP drafted the manuscript. All authors made refinements and approved the final manuscript.

## Funding

All costs including reimbursement of participants and open access publication fees were/will be covered by YN’s university budget.

## Conflict of Interest Statement

The authors declare that the research was conducted in the absence of any commercial or financial relationships that could be construed as a potential conflict of interest.
